# Large-scale prediction of outer-membrane multiheme cytochromes uncovers hidden diversity of electroactive bacteria and underlying pathways

**DOI:** 10.3389/fmicb.2024.1448685

**Published:** 2024-10-01

**Authors:** Arkadiy I. Garber, Kenneth H. Nealson, Nancy Merino

**Affiliations:** ^1^Biodesign Center for Mechanisms of Evolution, School of Life Sciences, Arizona State University, Tempe, AZ, United States; ^2^Department of Earth Sciences, University of Southern California, Los Angeles, CA, United States; ^3^Biosciences & Biotechnology Division, Lawrence Livermore National Laboratory, Livermore, CA, United States

**Keywords:** electroactive, extracellular electron transport, EET, multiheme cytochrome, MHC, porin

## Abstract

Multi-heme cytochromes (MHCs), together with accessory proteins like porins and periplasmic cytochromes, enable microbes to transport electrons between the cytoplasmic membrane and extracellular substrates (e.g., minerals, electrodes, other cells). Extracellular electron transfer (EET) has been described in multiple systems; yet, the broad phylogenetic and mechanistic diversity of these pathways is less clear. One commonality in EET-capable systems is the involvement of MHCs, in the form of porin-cytochrome complexes, pili-like cytochrome polymers, and lipid-anchored extracellular cytochromes. Here, we put forth MHCscan—a software tool for identifying MHCs and identifying potential EET capability. Using MHCscan, we scanned ~60,000 bacterial and 2,000 archaeal assemblies, and identify a diversity of MHCs, many of which represent enzymes with no known function, and many found within organisms not previously known to be electroactive. In total, our scan identified ~1,400 unique enzymes, each encoding more than 10 heme-binding motifs. In our analysis, we also find evidence for modularity and flexibility in MHC-dependent EET pathways, and suggest that MHCs may be far more common than previously recognized, with many facets yet to be discovered. We present MHCscan as a lightweight and user-friendly software tool that is freely available: https://github.com/Arkadiy-Garber/MHCscan.

## Introduction

**Figure 1:**
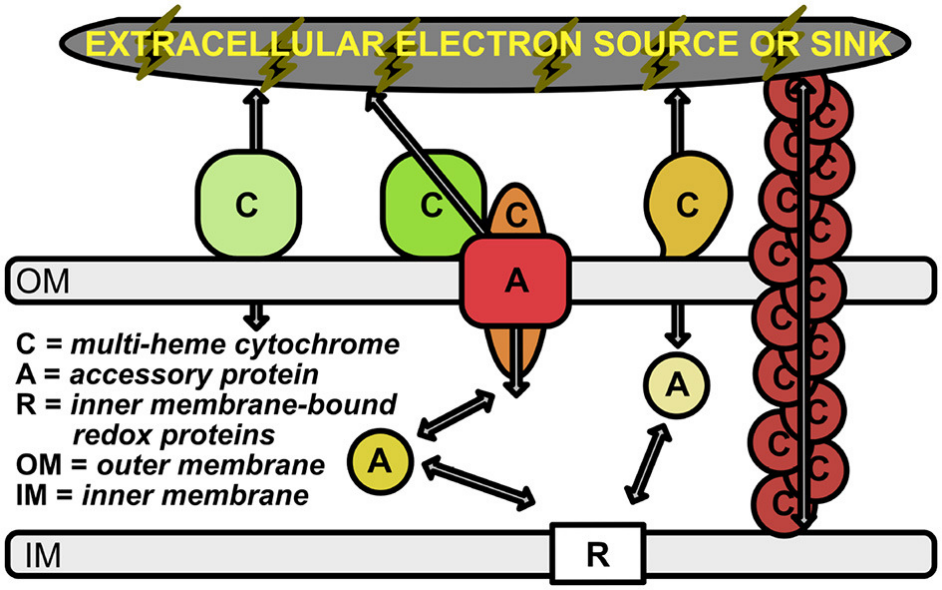
Schematic of electron transport across the periplasmic space and outer membrane. Outer membrane-bound multi-heme cytochromes are labeled with “C”. The multimeric OmcS cytochromes (shown in brown at the right of the figure) represent multi-heme cytochromes that are bound to the inner membrane, but extend out past the outer membrane. Accessory proteins are labeled with “A”; these represent porins and soluble periplasmic cytochromes that transport electrons from the inner membrane-bound electron carriers (labeled with “R”) to the outer membrane cytochromes. Arrows represent the flow of electrons.

The ability of cells to either donate or accept electrons from external insoluble substrates has been well-studied in several microbial groups [e.g., *Shewanella oneidensis* (Myers and Myers, 1992, 2002; Pitts et al., 2003; Hartshorne et al., 2009; Clarke et al., 2011; Kasai et al., 2015), *Geobacter sulfurreducens* (Shi et al., 2009; Merkley et al., 2015) *Rhodopseudomonas palustris* (Jiao and Newman, 2007; Bird et al., 2014), and *Ferroglobus placidus* (Smith et al., 2015)]. However, the importance of many of these microbe-mineral interactions remain hidden in the to-be-discovered intricacies of energy exchange and syntrophic interactions of the microbial world. Many of the known electrochemical interactions have recently been outlined and reviewed by Lovley and Holmes (2022); there are likely many more waiting to be discovered, as discussed below. Known mechanisms for EET include only a handful of protein families, many of which consist of multi-heme cytochromes (MHCs) that are localized to the periplasm and outer membrane of the cell (Shi et al., 2009; Richardson et al., 2012; Shi et al., 2014; Liu et al., 2014; He et al., 2017; Deng et al., 2018; Barco et al., 2015) (Figure 1). This is particularly true in the case of Gram-negative bacteria, and at least one Gram-positive species (*Thermincola potens*) is known to encode MHCs that link the cytoplasmic membrane with extracellular substrates through the cell wall (Carlson et al., 2012).

MHCs that are known to play a role in EET have been used as queries to search for homologous proteins and mechanisms in other microorganisms. For example, profile hidden Markov models (HMMs) have been compiled for known MHCs with EET function and are currently packaged in a bioinformatics tool called FeGenie to predict genetic potential for iron-cycling (Garber et al., 2020). However, many types of organisms are being discovered that are known to generate electrical currents (Logan et al., 2019), many of which do not appear to encode any of the related EET machinery. It is, thus, possible that these microbes produce MHCs with independently evolved EET pathways invisible to homology-dependent searches. With this in mind, we hypothesized that MHCs can be used to predict EET capability without the use of homology-dependent methods.

We searched the predicted proteomes of ~60,000 sequenced Bacteria and Archaea (from NCBI's RefSeq database) for gene neighborhoods encoding one or more MHC localized to the periplasm, outer-membrane, and/or extracellular space. This homology-independent approach (Supplementary Figure 1), revealed thousands of MHC-encoding gene neighborhoods, some predicted to have over a hundred heme-binding motifs, potentially involved in EET. Using FeGenie as a reference, we were able to identify novel MHCs for which gene markers are not yet publicly available. While FeGenie includes 14 protein families representing MHCs, sequence-based clustering of novel MHCs identified in our survey resulted in an additional 76 protein families encoding heme-rich (≥4 hemes) cytochromes that are localized to the periplasm, outer membrane, or extracellular space. Additionally, we found 42 novel protein families representing accessory genes, likely encompassing porins and soluble electron shuttles.

## Results and discussion

### Validation of the homology-independent approach

**Figure 2:**
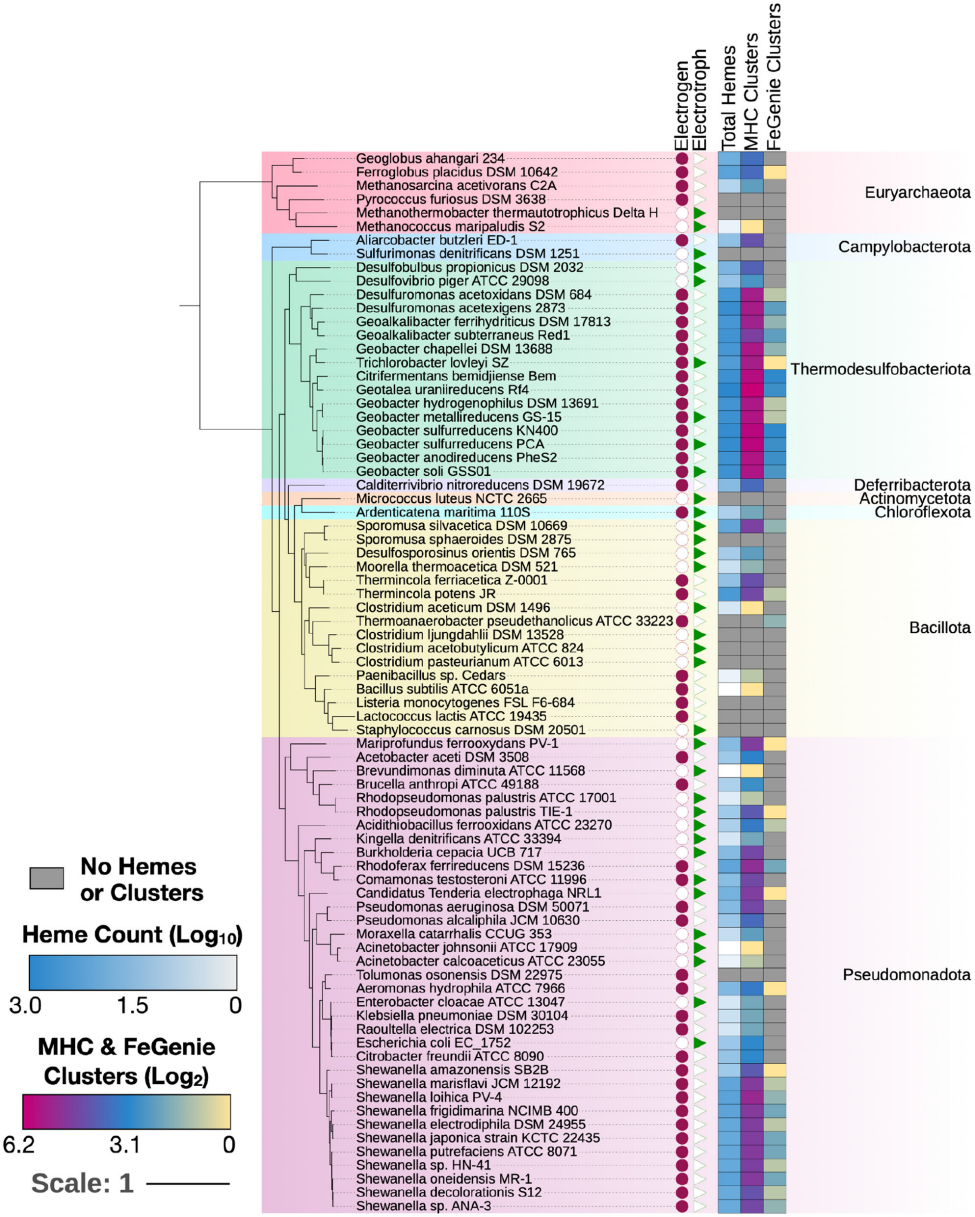
MHCs identified in 70 electroactive microbes as reviewed in Logan et al. (2019). In nearly all microbes, MHCScan identified more EET-related proteins than FeGenie. These EET candidates may represent novel mechanisms for electroactivity. A phylogenetic tree was constructed using GToTree and annotated using the Interactive Tree of Life (Letunic and Bork, 2021). Whether each microbe is an electrogen or electrotroph is denoted by the purple circles or green triangles to the right of the tree. The heatmap shows, per genome, the number of hemes, as well as MHC-encoding gene clusters potentially involved in EET. The number of MHC-encoding gene clusters identified with MHCScan (middle column) is generally higher than the number of gene clusters identified through the homology-dependent FeGenie software, suggesting new EET candidate genes.

To validate MHCscan, we used it to analyze a set of genomes from model organisms with characterized EET genes and pathways [e.g., *Rhodopseudomonas palustris* (Jiao and Newman, 2007), members of the *Shewanella* and *Geobacter* genera (Shi et al., 2012), and others]. All genomes analyzed in this benchmark are listed in Supplementary File 1, a select subset are listed in Table 1. Our criteria for the inclusion of gene neighborhoods potentially involved in EET were: (1) the presence of at least one MHC with a signal peptide; (2) the presence of at least one heme-rich (≥4 hemes) MHC; and, (3) the combined presence of at least 15 hemes within the entire multi-gene cluster. The exact parameters of the search can be tuned to include, for example, the single-heme porin-cytochrome Cyc2 or other outer-membrane cytochromes not matching the above criteria. Moreover, MHCscan will also report type IV aromatic pili (T4AP), which generally do not encode heme-binding motifs, but may nonetheless be involved in EET (Vargas et al., 2013). Our search with MHCscan accurately identified all known EET-related mechanisms in the subset of electroactive and iron reducing/oxidizing microbes. Moreover, within even these well-studied bacteria, we found additional gene neighborhoods with as-of-yet unnamed genes encoding multiheme cytochromes (Supplementary Figure 2, Supplementary Files 1, 2). We note that MHCs encoded by as-of-yet unnamed genes may have been described in previous research studies—nonetheless, appear as “hypothetical” in public repositories. We also used MHCscan to analyze genomes from 70 electroactive microbes included in the review by Logan et al. (2019). We compared predicted MHCs from MHCscan to EET-related genes identified through the homology-dependent software tool FeGenie. In these electroactive microbes, MHCscan detects more EET candidate genes than FeGenie (Figure 2), possibly illuminating a greater proportion of heme groups involved in facilitating extracellular electrical currents.

**Table 1:** GenBank assembly accessions and publications associated with the genomes used in validation of the homology-independent approach.

**Genome**	**GenBank assembly accession**	**Citation**
*Geobacter anodireducens*	GCA_014883105.1	Sun et al., 2014
*Gallionella capsiferriformans ES-2*	GCA_000145255.1	Emerson et al., 2013
*Ferrimonas balearica*	GCA_000148645.1	Nolan et al., 2010
Endosymbiont of *Riftia pachyptila*	GCA_000224455.2	NA
*Desulfuromonas* sp. TF 3336	GCA_000472285.1	Kim et al., 2014
*Shewanella colwelliana*	GCA_001735525.1	NA
*Archaeoglobus veneficus*	GCA_000194625.1	NA
*Candidatus* Syntrophoarchaeum caldarius	GCA_001766815.1	Laso-Pérez et al., 2016
*Ferroglobus placidus* DSM 10642	GCA_000025505.1	Anderson et al., 2011
*Candidatus* Methanoperedenaceae archaeon	GCA_003104905.1	Cai et al., 2018

### Survey of bacteria and Archaea

**Figure 3:**
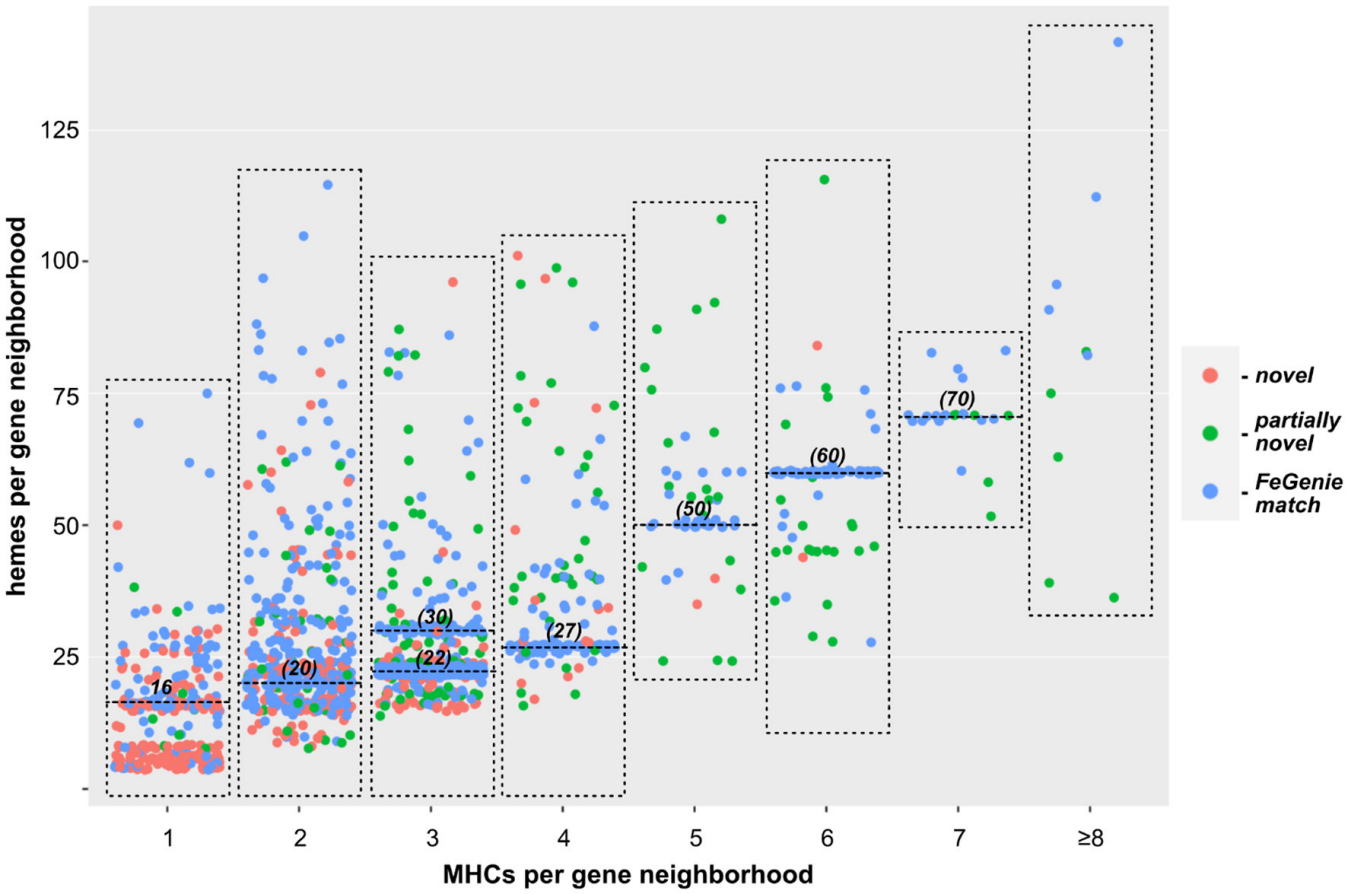
Scatterplot showing the relationship between the number of individual MHCs, and total number of hemes predicted within each gene neighborhood. Each dot represents a single gene neighborhood: those colored blue represent gene neighborhoods where all MHCs were predicted with FeGenie; green dots represent neighborhoods encoding at least one MHC not included in FeGenie's HMM library; red dots represent gene neighborhoods where not a single MHC in the gene neighborhood is currently included in FeGenie's HMM library. The high density of neighborhoods encoding multiples of ten hemes (horizontal lines at 20, 30, 50, 60, and 70 hemes per neighborhood) represent the decaheme cytochromes (MtrA/MtrC) encoded mostly in the *Shewanella* spp.; those encoding 22 and 27 hemes per neighborhood represent Mtr-encoding gene clusters in Vibrio and *Aeromonas* spp., which also encode pentaheme and/or diheme cytochromes.

We next turned our approach to all sequenced Bacteria and Archaea, down-sampled to reduce the number of over-represented genomes (e.g., *E. coli, Streptococcus* and *Salmonella* spp.). We identified 1,475 bacterial genomes (220 unique genera) and 75 archaeal genomes (35 unique genera), each with at least one gene neighborhood that encodes one or more clusters of MHCs. Most of these genomes encode only one such gene neighborhood; many encode a few, and a few genomes encode more than ten. In total, we identified over two thousand gene neighborhoods with potential involvement in EET (listed in Supplementary Files 1, 2 for Bacteria and Archaea, respectively). Of these, 204 gene neighborhoods encoded MHCs that can be identified using FeGenie's HMM library; 440 gene clusters were completely novel with no homologs present in FeGenie's library of HMMs (Figure 3). Species within *Vibrio, Shewanella, Geobacter, Geomonas*, and *Aeromonas* (e.g., known electroactive microbes) make up the majority of the lineages that we identified with our approach. These well-studied genera contain both the greatest diversity and the total number of MHC-encoding gene neighborhoods.

### Novel MHC families

There are 14 EET-linked MHCs represented within FeGenie's library of HMMs. Adding the MHCs that were identified by MHCscan, we identified an additional 118 protein families, each representing either a heme-rich MHC or accessory protein found within the cluster. Within this group of 118 novel HMMs, 76 represent MHCs that are predicted to bind five or more hemes. Notably, the greatest number of identified MHC-encoding gene neighborhoods were related to *Shewanella's* MtrCAB iron reductase complex (crystal structure in Edwards et al., 2020), in addition to DFE_0462 and DFE_0449, which encode MHCs in *Desulfovibrio ferrophilus* (Deng et al., 2018). It's possible that these pathways may be most efficient or most-easily transferred between bacteria via lateral gene transfer (LGT), as previously suggested for MtrCAB (Baker et al., 2021). Nonetheless, we uncovered many species and genera with no previous evidence for EET (Supplementary Files 1, 2) and suggest that EET is far more common than previously realized.

## Concluding remarks

Homology-dependent approaches that utilize sequence alignment (e.g., BLAST) and hidden Markov models (e.g., HMMER) allow for identification of evolutionarily conserved proteins. However, it's possible for certain structures and functions to arise independently. As an example, extracellular electron transport mechanisms are found across all reaches of the bacterial phyla; yet, only a handful of mechanisms are known. Our approach leverages the structural features and motifs of known EET-related enzymes to identify similar mechanisms in unrelated proteins, which may have evolved independently or diverged beyond where homology-dependent approaches can detect. The growing reportoire of software tools for *de novo* protein folding (e.g., AlphaFold3 and ESMFold) will also provide a powerful approach for the preparation of biochemical models for testing through *in vitro* and *in vivo* experimentation.

## Methods

### Genome acquisition

Over 100,000 genome assemblies were downloaded from NCBI's RefSeq database (release 209). We down-sampled the genome assemblies from well-studied, but over-represented, species (e.g., *Escherichia coli, Salmonella enterica*, and *Yersinia pestis*, etc.), and reduced the number of genomes to ~60,000.

### Workflow

The MHCscan pipeline, outlined in Supplementary Figure 1, starts with prediction of genes and corresponding protein sequences from genomes using Prodigal (Hyatt et al., 2010). If annotation was done beforehand, annotated genomes can be provided in the form of FASTA amino acid and GFF files. Phobius (Käll et al., 2004) is used to predict signal peptides and transmembrane helices. Heme c-binding motifs are predicted using FindMeHemes (https://github.com/Arkadiy-Garber/FindMeHemes). Output from Phobius and FindMeHemes is then processed to identify gene neighborhoods encoding multi-heme cytochromes that have signal peptides and appear to be periplasmic, outer membrane-bound, or secreted. We then removed the MHC gene clusters where the total number of predicted heme groups (across all proteins encoded within each cluster) falls below 15, an empirically derived number based on MHCs in known electroactive microbes.

### HMM development

To bring into light the novel MHCs potentially involved in EET, we used the identified protein sequences to generate hidden Markov models (HMMs). We performed clustering of identified MHC protein sequences using MMseqs2 (Steinegger and Söding, 2017). These clustered protein sequences were aligned using Muscle (Edgar, 2004), masked to remove positions consisting of mostly gaps, and used to generate hidden Markov models (HMMs) using HMMER (Johnson et al., 2010). HMMs were only generated for proteins families that were represented by at least three orthologs among the roughly 60,000 surveyed genomes. The only custom script used in HMM generated was masker.py, available here: https://github.com/Arkadiy-Garber/BagOfTricks.

## Data Availability

The original contributions presented in the study are included in the article/Supplementary material, further inquiries can be directed to the corresponding authors.
