# Supplementary material for: Large-scale prediction of outer-membrane multiheme cytochromes uncovers hidden diversity of electroactive bacteria and underlying pathways
Source: Front Microbiol. 2024 Oct 1;15:1448685. doi: 10.3389/fmicb.2024.1448685 (PMC11475568; doi:10.3389/fmicb.2024.1448685)
Supplement: Supplementary file 3 [file Data_Sheet_3.pdf]

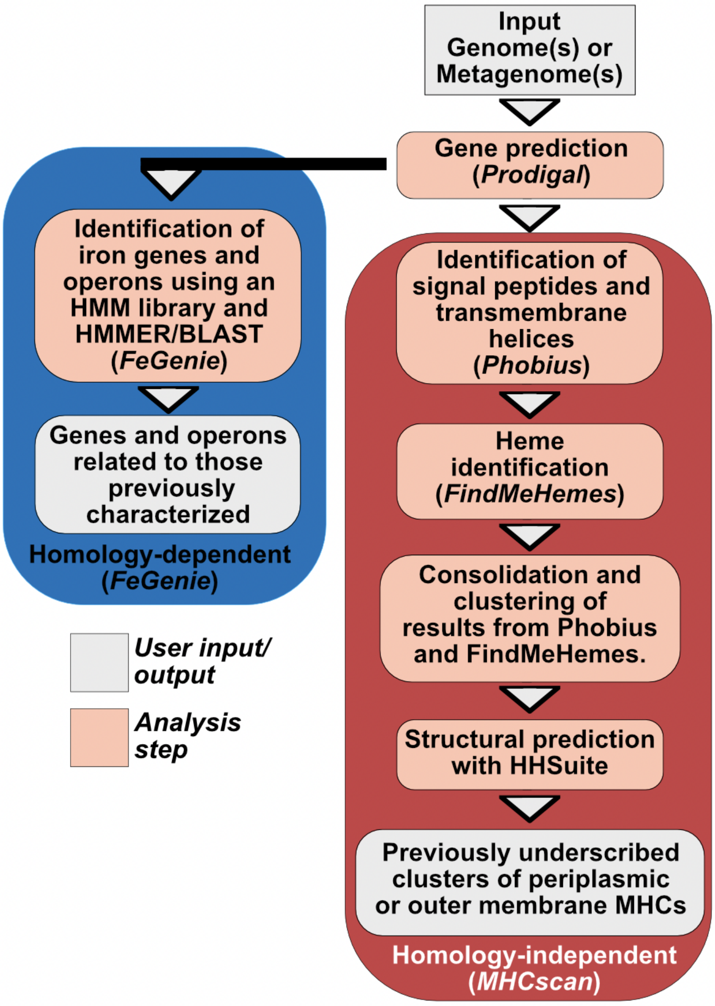

Supplemental Figure 1: Comparison of FeGenie's homology-dependent pipeline (left, light-blue) with our new novel homology independent approach (right, salmon-color). Both pipelines accept as input genome or metagenome assemblies.

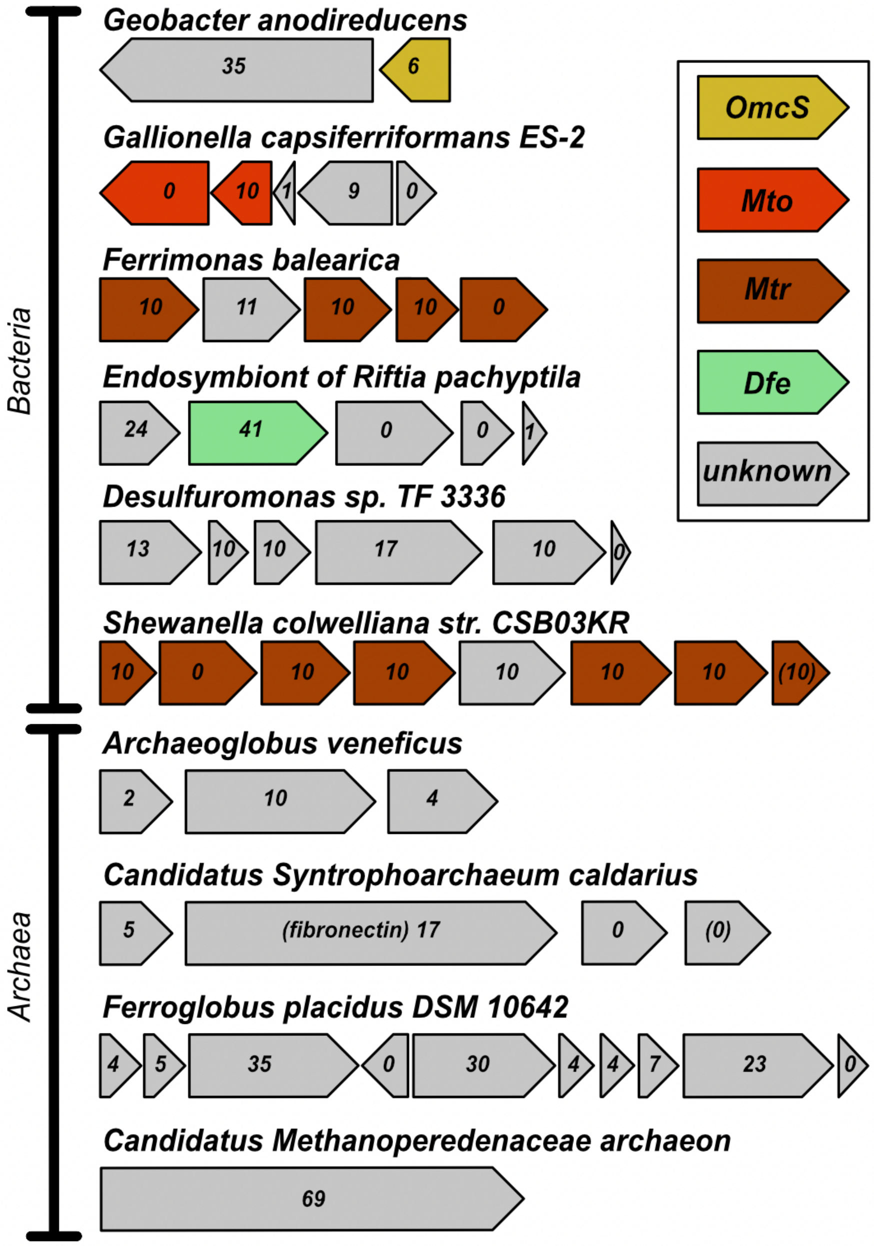

Supplemental Figure 2: MHC-rich gene neighborhoods/operons identified in various genomes in our survey. Genes are color-coded based on original characterization: yellow=OmcS (Qian et al., 2011), red=MtoAB (Liu et al., 2012), brown=MtrCAB (Pitts et al., 2003), green=DFE (Deng et al., 2018), gray=unannotated on RefSeq. Numbers within each gene indicates the number of heme-binding motifs predicted for the encoded protein.
